# Increased Cortical Thickness in Sports Experts: A Comparison of Diving Players with the Controls

**DOI:** 10.1371/journal.pone.0017112

**Published:** 2011-02-16

**Authors:** Gaoxia Wei, Yuanchao Zhang, Tianzi Jiang, Jing Luo

**Affiliations:** 1 Key Laboratory of Mental Health, Institute of Psychology, Chinese Academy of Sciences (CAS), Beijing, People's Republic of China; 2 School of Life Sciences and Technology, University of Electronic Science and Technology of China, Chengdu, People's Republic of China; 3 National Laboratory of Pattern Recognition, Institute of Automation, Chinese Academy of Sciences, Beijing, People's Republic of China; French National Centre for Scientific Research, France

## Abstract

Sports experts represent a population of people who have acquired expertise in sports training and competition. Recently, the number of studies on sports experts has increased; however, neuroanatomical changes following extensive training are not fully understood. In this study, we used cortical thickness measurement to investigate the brain anatomical characteristics of professional divers with extensive training experience. A comparison of the brain anatomical characteristics of the non-athlete group with those of the athlete group revealed three regions with significantly increased cortical thickness in the athlete group. These regions included the left superior temporal sulcus, the right orbitofrontal cortex and the right parahippocampal gyrus. Moreover, a significant positive correlation between the mean cortical thickness of the right parahippocampal gyrus and the training experience was detected, which might indicate the effect of extensive training on diving players' brain structure.

## Introduction

Since Chase and Simon (1973) proposed their influential theory of expertise, cognitive approaches have become more common in the study of expertise, which is the growth of specialist knowledge and skills as a result of effortful experience. Undoubtedly, extensive practice is required to establish expertise, whether for a language or for motor learning. Previous studies have shown that extensive training, such as meditation practice [1], [2], [3], language learning [4] or career experience [5], could induce neuroplasticity, which refers to brain's ability to change its structure and function throughout an individual's lifetime [6]. Recently, many studies have focused on musicians' brains, which have revealed that significant structural change was induced by instrumental practice [7], [8]. Among the participants in the studies exploring the neuroplasticity associated with practice or training, the expert population obviously provides a rich source of empirical evidence on the true potential of human achievement [9], [10], since this highly trained population exhibits a number of differences including a reduction of the variability of repeated skilled movement [11], reductions in muscle activation [12], and better information-processing performance relevant to their skills or knowledge [13]. However, both functional and anatomical studies of experts' brain are not as abundant as those of learning-dependent neuroplasticity that adopt a longitudinal paradigm. Although some researchers began to be interested in experts' perceptual-cognitive approach at the behavioural level [14], the number of reports on experts' brains is very limited.

Studies of animals and humans have demonstrated that motor training with a certain intensity and frequency is associated with changes in the brain structure. Van-Praag and his colleagues (1999) found that running increases neurogenesis in the dentate gyrus of the hippocampus, a brain structure that is important for memory function [15]. Another study confirmed that maternal running during pregnancy resulted in a significant increase in the expression of BDNF mRNA, enhanced hippocampal cell survival, and improved the short-term memory capability of rat pups, as compared to the control group [16]. Exercise also alters the morphology of the dentate granule cell dendrites of adult rats [17]. Abundant evidences from animal models suggest that exercise could be regarded as an important intervention to improve brain health and induce neuroplasticity [18]. Additionally, studies on humans revealed that the brain structures of experts in playing basketball, playing golf or practicing judo are different from that of the general people [8], [19], [20], [21], [22], [23]. However, the results obtained by these studies lack consistency. The neuroanatomical changes following extensive training are not fully understood.

Sports experts are usually engaged in motor skill learning for a long period of time and can display their extraordinary skill in stressful situations. The majority of professional sports, which are performed by well-trained individuals, usually are executed in a dynamic, ever-changing environment, under conditions of extreme stress where the limits of human behaviour and achievement are being continually challenged and extended [14]. Because of high frequency and high intensity training, and super achievement in motor skills, professional athletes might represent typical population that has acquired certain motor expertise. Among these people, diving players are kind of sports experts who are engaged in performing complex and precise skills. The object of diving is to produce the intended body parabolic trajectory with multiple twists and somersaults in accordance with the law of conservation of angular momentum. It possesses some characteristics as follows: 1) Air awareness towards their self-movements is greatly emphasized since control of rotation is demanded during the execution of diving. 2) Diving is a typical example for closed-loop control movements with much complexity, so spatial navigation on movements is highly needed to achieve the best possible diving. 3) Precise kinesthesis also plays an important role for accomplishing perfect movements. 4) Exact position of hands, arm, legs and feet are key elements for executing the submovements such as the approach, the flight and the entry. Hence, diving is a typical example of movements which is performed against air resistance under the influence of gravity, so spatial perception of body position is greatly demanded.

In previous study, voxel-based morphometry (VBM) was used to examine the difference of grey matter following extensive training between diving players and the controls [24]. However, the smoothing step of VBM neglects the anatomical relationships across the folded cortical surface [25], which might reduce the sensitivity to detect significant effects. Cortical thickness, a more direct and biologically meaningful measurement, has been proven to be sensitive of neurodevelopmental and pathological changes and a reflection of the architecture of the cerebral column [26], [27], [28]. Therefore, cortical thickness was applied in this study to further examine the structural differences between diving players and the controls. Given that diving is closed-loop motor skills attaching importance to spatial information processing of body movement, changes are anticipated in parahippocampal gyrus, which was shown to be involved in acquiring spatial information.

## Results

**Figure 1:**
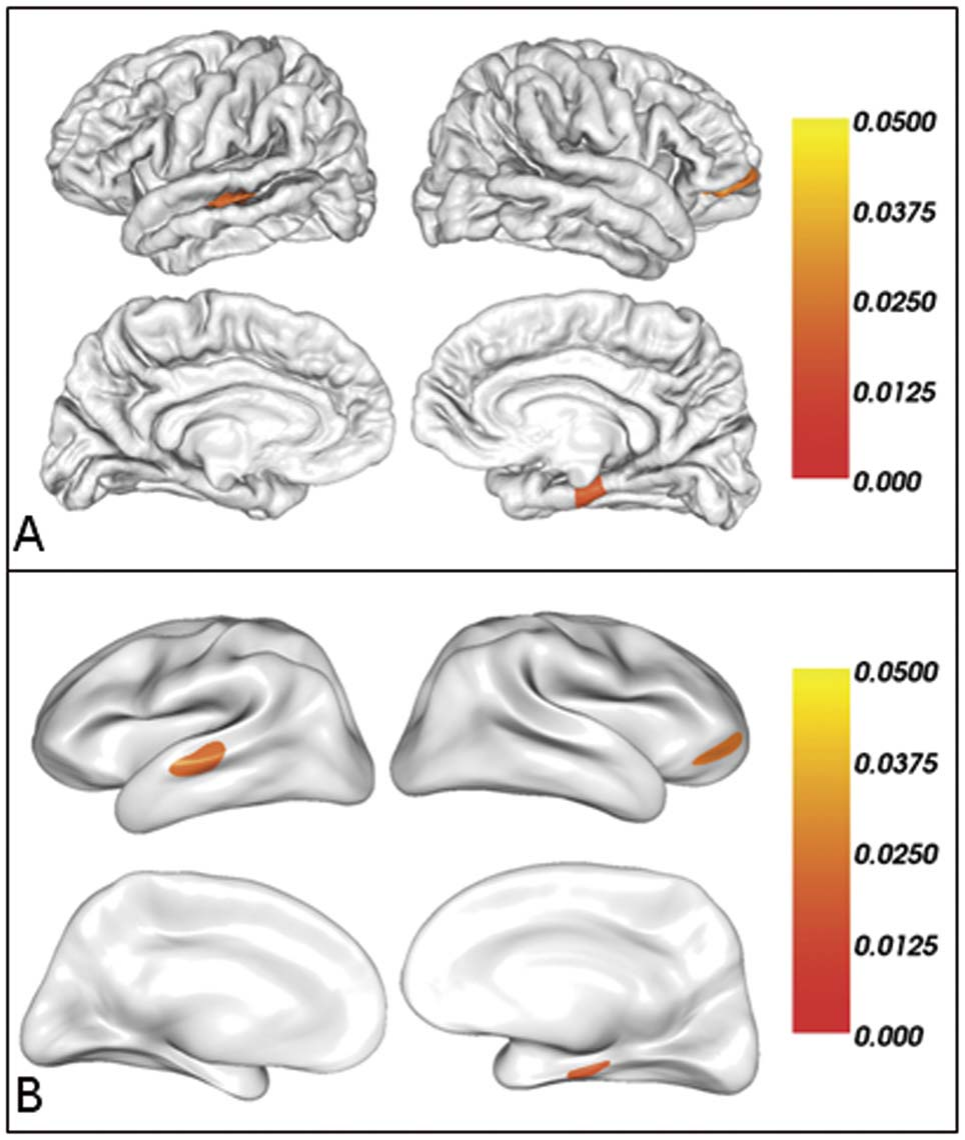
Brain regions with increased cortical thickness in expert compared with novices after a correction for multiple comparisons (P<0.05, the cluster-based RFT correction) on the pial surface. The color bar indicates the cluster-wise *p*-value after the correction for multiple comparisons.

**Figure 2:**
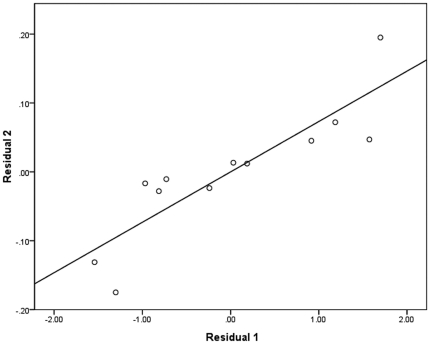
Scatter plot of two residuals. It showed that the cortical thickness is increased associated with more years of training (*r* = 0.87; *p* = 0.0001). Residual 1: residuals were obtained from regression of training against age and education. Residual 2: residuals were obtained from regression of cortical thickness against age and education.

Compared with non-athletes group (NG), athletes group (AG) had significantly increased cortical thickness. We found three regions of difference with thresholds of p<0.05 (RFT corrected) and cluster size ≥100 vertices (Fig 1). These regions included the left superior temporal sulcus (p = 0.0174; cluster size  = 1350 vertices), the right orbitofrontal cortex (p = 0.0217; cluster size  = 694 vertices) and the right parahippocampal gyrus (p = 0.0139; cluster size  = 738 vertices). Adding sex, age and brain size as covariates did not appreciably alter our results (Fig S1 in Supplementary Materials), although the significant level and cluster size of each cluster somewhat decreased after controlling these effects. For visualisation, regions of difference were projected onto the pial and inflated surfaces of the average template. In addition, a significant positive correlation was detected between the cortical thickness of the right parahippocampal gyrus and the years of training involved in motor practice in AG after controlling for age and education (r = 0.87, p = 0.0001) (Fig 2), while no significant correlations were observed between the other two regions and the training experience.

## Discussion

In this study, we used a surface-based approach to explore the difference in the cortical thickness between a sport expert group and a novice group that were matched for gender and age. The findings of this study showed that compared with the novices, divers had increased cortical thickness in the left superior temporal sulcus, the right parahippocampal gyrus and the right orbitofrontal cortex. Additionally, the years of training experience was significantly correlated with the mean cortical thickness of the right parahippocampus gyrus.

Current study in the parahippocampal gyrus supports the idea that this neuroanatomical structure may be closely related to acquiring skills during diving. The results described in this study demonstrated the difference between the diving players and matched controls, and revealed the positive correlation between increasing cortical thickness and the years of training. We propose that these results might reflect the effect of extensive skill learning, which is certainly a feature distinguishing players from normal controls, upon the parahippocampal gyrus. Previous morphometrical studies on humans consistently found the positive correlation between musician status and grey matter volume [8], between the meditation experience and the cortical thickness [29], between the taxi driving experience and the volume of the right posterior hippocampus [30], between knowledge learning and the grey matter of posterior hippocampus [31], which suggested the experience/learning induces the plastic changes of particular brain structures. Hence, according to our findings in the parahippocampal gyrus, it implied that long-term skill learning contributes to the plasticity of the parahippocampal gyrus. Although there is no direct evidence to infer that the change in the parahippocampal gyrus is related to a certain motor or mental task, it is reasonable to propose that it might be associated with expertise, especially the spatial information processing in view of the role of this mental processing in diving. Several studies have described the involvement of the parahippocampus in retrieving spatial information [32], [33]. Neuropsychological and neuroimaging studies also suggested the parahippocampus was involved in acquiring spatial information [34], [35], [36]. Further functional studies found spatial memory deficits were induced in the patients with lesions of parahippocampal gyrus [37], [38], [39]. Moreover, our previous functional study also found that divers had greater activation than the novices in the parahippocampus when performing diving imagery, which involved the same subjects as the present study [40]. The consistent findings between functional and structural imaging in the parahippocampus contributed to the inference that the change in parahippocampal gyrus is associated with increasing expertise. But it is not clear that this neuroanatomical structure is related to the general expertise or motor-related expertise. In the opinion of the authors, the findings pertaining to this should be interpreted more cautiously since there is no behavioural evidence to indicate the role of parahippocampus in spatial information processing.

Our results for the superior temporal sulcus (STS) are of particular interest. This region is known to play an important role in the perception of biological motion [41] and has been suggested to be responsible for using a body shape-based model to process biological movement [42]. The increased thickness in the STS in the athlete group may indicate that the athletes are much better at perceiving movements performed by others, even when insufficient perceptive information is provided. Additionally, several researchers have argued that this region is not only related to biological motion but also responds to the evaluation of the intentions behind other people's actions, in particular, to understand the relationship between the observed motion and the structure of the surrounding environment [43], [44]. This type of understanding seems to be crucial for divers since they always learn movements through observing other divers' performances and by comparing others' movements with their own intentions.

The orbitofrontal cortex (OFC) has been reported to regulate the planning of behaviours associated with reward and punishment [45] and to mediate subjective hedonic experiences [46]. Evidence from patients with injured OFCs has shown that damage to this region leads to a pattern of disinhibited behaviour [47]. Although it is unclear why the increased thickness of the OFC in the athlete group was observed, it might be inferred that athletes have good regulative ability with respect to rewards (such as medals and honours) and punishment (such as failure in the competition).

Our study revealed that the sports experts' brains are quite different from those of the general population, and the different brain region is associated with extensive training. However, the correlation between the training duration and the thickness of the STS and the OFC did not reach statistical significance. One reason might be the small variation in the number of years spent training. Although we cannot completely rule out the effect of maturation and innate composition, we infer that this change is associated with training or practice. Abundant evidence consistently supports the fact that extensive training for a specific task induces corresponding structural changes [48], [49].

In summary, we investigated the sports experts' brain structure by comparing an expert group with a novice group. Nevertheless, we still need to pay more attention to the following points. First, determining which factor (long-term dedicated practice or innate differences) contributes to the structure difference needs to be further investigated. Perhaps a longitudinal study on the same expert group in developing motor skills might be needed to elucidate whether the difference is induced by reorganisation or redistribution. Second, it is necessary to investigate the neuroanatomical structures being responsible for expertise in general and task-related expertise respectively in the future. One approach is to collect sports experts' cognitive or mental performance of a certain expertise, and then examine the correlation between brain structure and the behavioural indicators. This would be helpful to detect the mechanisms underlying a certain expertise and also provide a window to examine neuroanatomical plasticity following extensive training. The other approach is to make reasonable experiment design to detect task-related expertise. For example, we could compare two experts groups sharing very similar components of expertise except only one difference/variable, with the control group, which aimed to extract this different component in expertise. Third, we suggest that sports experts' brains may offer a new model in the field of cognitive neuroscience for functional or anatomical studies.

## Materials and Methods

### Ethics Statement

The written informed consent from their parents was obtained, and the study “functional and anatomical plasticity of sports experts” was approved by the Institutional Review Board of the Beijing MRI Center for Brain Research and was performed in accordance with the ethical standards laid down in the 1964 Declaration of Helsinki. The ethics committee specifically approved all of the procedures of this study. Before the scans were taken, all subjects delivered the volunteer screening forms to the Beijing MRI Centre for Brain Research to exclude any subjects who had a history of hearing or vision problems, physical injury, seizures, metal implants, and head trauma with loss of consciousness, or pregnancy.

### Subjects

In this study, AG comprised 12 professional diving players with top-level skills (6 females and 6 males). NG was matched for age, educational level (confirmed by t-test) (see table 1 for detailed information) and gender (6 females and 6 males) and was composed of healthy subjects who were not involved in any extensive physical training or professional experience. There was no significant differences in age (*t* (11)  = -0.60; *p* = 0.56) and education (*t* (11)  = −0.30; *p* = 0.77) between AG and NG. All of the subjects were right-handed and were medically and neurologically stable. No subjects had any lifetime histories of substance dependence.

**Table 1:** Demographic characteristics of the samples.

	Athletes group(n = 12)	Non-athletes group(n = 12)
Age (year)Education (year)	14.58 (1.68)*7.75 (1.82)*	14.92 (1.38)*7.92 (1.38)*
Average practice time per day (hr)	6.54 (0.38)	N/A
Duration of practice (year)Age of commencement (year)	10.12 (0.86)5.33 (0.98)	N/AN/A

Data are mean (SD).

*no significant difference between groups *(p*>0.05).

### MR Scanning

High-resolution anatomical images of the whole brain were acquired on a 3-tesla Trio system (Siemens, Erlangen, Germany) with 12-channel head matrix coil using a magnetisation-prepared rapid-acquisition gradient echo sequence. The following parameters were used for the volumetric acquisition: TR  = 2530 ms, TE  = 3.37 ms, flip angle  = 7 degrees, slice thickness  = 1.33 mm, FOV  = 256 mm, 512×512- pixel matrix. The voxel size was 0.5×0.5×1.33 mm. The scan time for the T1-weighted sequence was 486 s, and the scan was conducted at the end of an fMRI session. During the scanning, each subject reclined in a supine position on the bed of the scanner and was asked to lie still during the imaging time. A foam head holder and padding were placed around the subject's head. In addition, headphones were provided to block background noise.

### Preprocessing

Each scan was processed using FreeSurfer (http://surfer.nmr.mgh.harvard.edu/) using the volume and surface pipeline [50], [51]. Starting from the segmentation of the white matter and the tessellation of the grey/white matter boundary, an initial surface was obtained after automated topological correction. This surface was used as the initial shape for the deformable model that was used to reconstruct the pial surface. When all of the surfaces had been reconstructed, the cortical thickness was computed. The thickness was measured in the native space of each subject. The thickness was defined at each point on the pial surface (as well as its counterpart on the grey/white matter surface because of the one-to-one correspondence) as the mean of the two shortest distances [52]; one was from the point on the pial surface to the grey/white surface, and the other was from the point on the grey/white matter surface to the pial surface. To compare cortical thicknesses point by point and to visualise the statistical results, the establishment of point correspondence across subjects in a standard surface-based coordinate system was required. Surface-based registration was used to build an average template, and all of the individual reconstructed cortical surfaces were aligned to this template [53]. Then, the cortical thickness data were resampled for each subject. Prior to statistical analysis, a heat kernel with a 30-mm width was used to smooth the cortical thickness maps to increase the signal-to-noise ratio and to improve the ability to detect morphometric variations [54].

### Statistical Analysis

Statistical analysis was performed at every vertex across all subjects. Two-sample t-tests were used to test for statistically significant differences in cortical thickness at homologous vertices. The threshold *p*≤0.001 was used to define clusters, and only clusters with a minimum of 100 points were reported. Then, a corrected cluster-wise p-value was obtained using random field theory (RFT) [55]. The level of significance for clusters was set at *p*<0.05 after multiple comparison correction. Moreover, the mean cortical thickness of the regions with significant differences were obtained for AG and the relationship between the mean cortical thickness and the years of training experience in AG were studied by the Pearson correlation coefficient.

## Supporting Information

Figure S1 Brain regions with increased cortical thickness in expert compared with novices after controlling for age, sex and brain size. For cluster 1, p = 0.0327 (RFT corrected) and cluster size  = 910; for cluster 2, p = 0.0957 (RFT corrected) and cluster size  = 236; for cluster 3, p = 0.0344 (RFT corrected) and cluster size  = 662.
(DOC)
