# Supplementary figures and images for: Increased Cortical Thickness in Sports Experts: A Comparison of Diving Players with the Controls
Source: PLoS One. 2011 Feb 16;6(2):e17112. doi: 10.1371/journal.pone.0017112 (PMC3040218; doi:10.1371/journal.pone.0017112)

Fig. S1


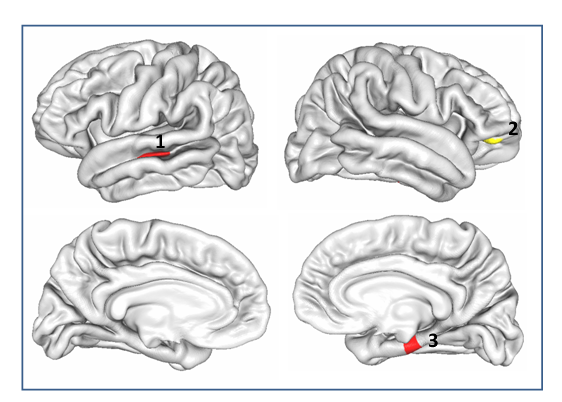

Supplement: Figure S1 — Brain regions with increased cortical thickness in expert compared with novices after controlling for age, sex and brain size. For cluster 1, p = 0.0327 (RFT corrected) and cluster size = 910; for cluster 2, p = 0.0957 (RFT corrected) and cluster size = 236; for cluster 3, p = 0.0344 (RFT corrected) and cluster size = 662. (DOC) [file pone.0017112.s001.doc]
